# Point-Prevalence Survey of Antimicrobial Use and Healthcare-Associated Infections in Four Acute Care Hospitals in Kazakhstan

**DOI:** 10.3390/antibiotics13100981

**Published:** 2024-10-17

**Authors:** Yuliya Semenova, Aizhan Yessmagambetova, Zaure Akhmetova, Manar Smagul, Akniyet Zharylkassynova, Bibigul Aubakirova, Kateryna Soiak, Zhanar Kosherova, Ainur Aimurziyeva, Larissa Makalkina, Ainur Ikhambayeva, Lisa Lim

**Affiliations:** 1 Department of Surgery, Nazarbayev University School of Medicine, Astana 020000, Kazakhstan; zbeysenova@nu.edu.kz; 2 National Center of Public Healthcare, Astana 010000, Kazakhstan; a.yesmagambetova@hls.kz (A.Y.); m.smagul@hls.kz (M.S.); dpiz@hls.kz (A.Z.); 3 Ministry of Health of the Republic of Kazakhstan, Astana 010000, Kazakhstan; z.akhmetova@dsm.gov.kz; 4 WHO Country Office in Kazakhstan, Astana 020000, Kazakhstan; aubakirovab@who.int (B.A.); soiakk@who.int (K.S.); 5 Nazarbayev University School of Sciences and Humanities, Astana 010000, Kazakhstan; ainur.aimurziyeva@nu.edu.kz; 6 Department of Clinical Pharmacology, Astana Medical University, Astana 010000, Kazakhstan; makalkina.l@amu.kz (L.M.); ikhambayeva.a@amu.kz (A.I.); 7 Nazarbayev University Graduate School of Public Policy, Astana 010000, Kazakhstan; lisa.lim@nu.edu.kz

**Keywords:** antimicrobial use, healthcare-associated infections, point-prevalence survey, acute-care hospital, Kazakhstan

## Abstract

Background/Objectives: Few studies have examined the prevalence of healthcare-associated infections (HAIs) and antimicrobial use (AMU) in acute care hospitals in Kazakhstan. This study aimed to address this gap by conducting a point-prevalence survey (PPS) of HAIs and AMU, as well as evaluating hospital antibiotic consumption via internationally recognized methodologies. Methods: PPS was conducted in four acute care hospitals in Kazakhstan on 11 May 2022, following the methodology of the European Center for Disease Prevention and Control, and included 701 patients. Antibiotic consumption in the same hospitals was assessed via the Global Antimicrobial Resistance and Use Surveillance System methodology. Results: HAIs were observed in 3.8% of patients (27/701), with intensive care unit wards accounting for 48.1% of these cases (13/27). *Pseudomonas aeruginosa* was the most frequently identified pathogen (5 out of 14 documented cases, 35.7%). Resistance to carbapenems was the most common resistance, followed by resistance to glycopeptides and third-generation cephalosporins. The rate of AMU was 38.2%, with an average of 1.37 antibiotics administered per patient. Surgical prophylaxis lasting more than one day was the most common indication for antimicrobial prescription (44.8%). Ceftriaxone and cefazolin are the most commonly used antibiotics. Conclusions: The results of this study are important for understanding the current situation in Kazakhstan and for informing national antimicrobial stewardship and infection control strategies.

## 1. Introduction

Antimicrobial resistance (AMR) is one of the major global health challenges today because of the increasing prevalence of resistant strains and the lack of novel antimicrobial classes. Antibiotics constitute the bulk of antimicrobial agents, and AMR most significantly impacts this class [1]. The overuse or misuse of antibiotics is the leading cause of the growing AMR crisis, highlighting the urgent need for antimicrobial stewardship (AMS) strategies aimed at promoting the responsible use of antibiotics. Understanding current prescription and usage practices is the cornerstone of AMS strategies, as it provides important insights into areas where interventions are needed [2]. Since most antibiotics are administered at the healthcare system level, there is a significant body of international research investigating antimicrobial use in hospital facilities [3].

Healthcare-associated infections (HAIs) are another emerging global health threat closely linked to AMR. Common HAIs include surgical-site infections, urinary tract infections, pneumonia, gastrointestinal infections, and bloodstream infections [4]. According to the Centers for Disease Control and Prevention (CDC), an estimated 687,000 patients acquire HAIs in hospital settings in the USA each year (1 in 31 patients), with as many as 72,000 of these patients dying as a result (0.3%) [5]. In Australia, one in ten patients in acute care hospitals has at least one HAI, and the prevalence of multidrug-resistant organisms is 10.3% [6]. In the European Union (EU) and European Economic Area, the prevalence of HAIs is estimated to be 7.1%, ranging from 3.1% to 13.8% depending on the country [7]. Although high-income countries have shown a declining trend in HAIs due to improved prevention and control measures, the prevalence of HAIs in low- and middle-income countries (LMICs) is likely higher due to limited resources, inadequate infection control practices, and a lack of prioritization [8].

The Republic of Kazakhstan (hereafter “Kazakhstan”) is an upper-middle-income country that gained independence in 1991 following the dissolution of the Union of Soviet Socialist Republics. The country inherited the Semashko model of healthcare, characterized by a well-established network of hospital facilities and a high uptake of hospital services. Although Kazakhstan has initiated a series of healthcare reforms, many features of the Semashko model persist today [9]. Several studies have investigated antibiotic consumption in hospital facilities and reported a growing trend [10,11]. However, few studies have examined the prevalence of HAIs, especially those utilizing internationally recognized methodologies that yield comparable results. To address this gap, this manuscript reports findings from a point-prevalence survey (PPS) on antimicrobial use and HAIs in four acute care hospitals in Kazakhstan conducted via the European Center for Disease Prevention and Control (ECDC) methodology [7]. These findings are supplemented by an analysis of antibiotic consumption in the same hospitals, which is based on the methodology of the Global Antimicrobial Resistance and Use Surveillance System (GLASS) of the World Health Organization (WHO) [12]. The results of this study are important for understanding the current situation in Kazakhstan and for informing national AMS and infection control strategies.

## 2. Materials and Methods

This cross-sectional study was conducted in two distinct stages. In the first stage, a PPS was implemented in 2022 in four acute care hospitals in Kazakhstan, following the methodology of the ECDC [7]. In the second stage, data analysis on antibiotic consumption in the same hospitals for the same year was carried out. Both stages contribute to understanding antimicrobial consumption and HAIs in acute care hospitals in Kazakhstan.

### 2.1. Study Hospitals

**Figure 1:**
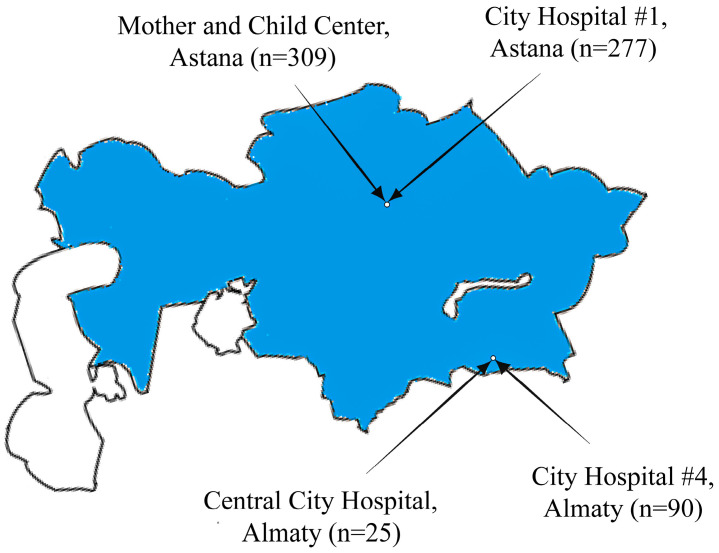
Map of Kazakhstan indicating the locations of hospitals included in the study and their respective contributions to the overall sample size.

Four hospitals were identified for the study, two located in Astana and two in Almaty, representing geographic northern and southern Kazakhstan, respectively. The Central City Hospital in Almaty is a secondary-level hospital with 553 beds, including 25 ICU beds. City Hospital #4, also in Almaty, is another secondary-level hospital with 470 beds, including 30 ICU beds. City Hospital #1 in Astana is a secondary-level hospital with 642 beds, including 25 ICU beds. The Mother and Child Center in Astana is a tertiary-level hospital with 500 beds, including 33 ICU beds. For ethical considerations, no comparisons between hospitals are made, and data from all hospitals are presented in a consolidated manner. Figure 1 shows the locations of the hospitals under study and their contributions to the overall sample size.

### 2.2. Point-Prevalence Survey of Antimicrobial Use and Healthcare-Associated Infections

The methodology outlined in version 5.3 of the protocol for the “Point Prevalence Survey of Healthcare-Associated Infections and Antimicrobial Use in European Acute Care Hospitals” [7] was strictly followed. The survey was conducted on a single day, 11 May 2022, and included all patients admitted to the hospital before or at 8:00 a.m. who had not been discharged on the day of the survey. Patients of all ages admitted to any type of ward present in the hospital were included. Patients receiving same-day treatment or surgery, those seen in the outpatient departments, those in the emergency room, and outpatient dialysis patients were excluded from the survey [7]. The total sample size for this study was 701 patients.

Patient files were reviewed to extract information on antimicrobial use and HAIs. The data collection form was filled out for each eligible patient (including those not receiving antimicrobials and not presenting with HAIs) and covered socio-demographic information, ward type, presence of risk factors (prior surgery, catheters, etc.), antimicrobial use, and severity of underlying disease. Antimicrobial use was recorded if at least one antimicrobial was being administered at the time of the survey, except for surgical prophylaxis, which was included if it was administered within 24 h prior to the survey. Antivirals and antimicrobials for the treatment of mycobacteria were not taken into consideration. An HAI was defined as an active infection at the time of the survey or a past infection for which the patient was still receiving treatment on the survey date, and which was not present at the time of admission. The ECDC PPS Protocol, version 5.3, was consulted to determine whether study participants met the definition of an HAI. Microbiological results were collected only if they were available on the survey date, and active HAI was defined according to the case definition stated in the ECDC protocol [7]. The susceptibility phenotype was classified as susceptible, intermediate, resistant, or unknown. In this manuscript, only resistant strains are presented.

All surveyors were staff members of the National Center for Public Health (NCPH), which coordinated and carried out this PPS with technical assistance from the WHO country office. The NCPH organized a training session for the participating hospitals prior to data collection. The collected data were initially entered into forms based on the protocol’s version 5.3 and subsequently transferred into a computer database, which was Helics.Win.Net application, version 4.1.0.

### 2.3. Antimicrobial Consumption in Hospitals

To analyze hospital antimicrobial consumption, data from SK-Pharmacia, the sole supplier of pharmaceuticals to hospital facilities in Kazakhstan, were accessed via a database maintained by Vi-ORTIS, a pharmaceutical market research company [13]. Data on systemic antibacterials (J01 code) from the Anatomical Therapeutic Chemical (ATC) classification system were extracted for the hospitals included in the PPS, covering the period from 1 January to 31 December 2022. The extraction was disaggregated by the ATC5 level and included details such as the active ingredient(s), dosage form, route of administration, number of preparations per package, and number of packages administered. The Vi-ORTIS database is commonly used for pharmacoepidemiological research [14].

The extracted data were entered into the Excel template from the Global Antimicrobial Resistance and Use Surveillance System (GLASS) guide for national surveillance systems monitoring antimicrobial consumption in hospitals [12]. This approach enables the calculation of defined daily doses (DDDs) per 100 patient-days for each ATC5 code [15]. The AWaRe classification of antibiotics [16] was then used to categorize all antibiotics into “Access”, “Watch”, and “Reserve” groups.

### 2.4. Statistical Analysis

The Statistical Package for the Social Sciences (SPSS) software, version 24 (Armonk, NY, USA: IBM Corporation), was used for data analysis. The distribution of continuous variables, such as patient age, was assessed via the Kolmogorov–Smirnov test and graphically via histograms and Q–Q plots. Since the distribution deviated from normal, continuous variables are presented as medians with first and third quartiles (Q1 and Q3), and between-group comparisons were performed via the Mann–Whitney U test. Categorical variables are presented as frequencies (N) and percentages (%), and Pearson’s chi-square test or Fisher’s exact test was utilized for between-group comparisons. The significance level for all the statistical tests was set at 0.05.

Hospital wards were classified into three broad categories: medical, surgical, and ICU. Rehabilitation, endocrinology, hematology, rheumatology, neonatology, nephrology, and general medicine wards were categorized as “medical”. The neurosurgery, traumatology, orthopedics, burns, urology, obstetrics, gynecology, vascular surgery, and general surgery wards were categorized as “surgical”. Mixed ICU, medical ICU, and pediatric ICU wards were grouped as “ICUs”.

## 3. Results

### 3.1. Study of Antimicrobial Use and Healthcare-Associated Infections in Acute Care Hospitals

Antimicrobials were used in 268 of the 701 hospitalized patients (38.2%). Patients who received antibiotics were significantly older than those who did not (median age 42.0 years vs. 32.0 years, respectively). There was also a significant difference in sex distribution: in the antibiotic group, most patients were male, whereas in the nonantibiotic group, most patients were female. Most patients receiving antibiotics were treated in surgical wards (56.3%), whereas the majority of those in the nonantibiotic group were treated in medical wards (50.3%). In intensive care units, antibiotics were administered to 69 out of 86 patients (80.23%) (Table 1).

Overall, 268 patients received a total of 368 antibiotic prescriptions, with an average of 1.37 antibiotics per patient. In total, 168 patients (62.7%) received one antibiotic, 90 patients (33.6%) received two antibiotics, and 10 patients (3.7%) received three antibiotics. Ceftriaxone was the most commonly prescribed antibiotic, and was administered to 9 pediatric patients and 99 adult patients. Cefazolin was the second most commonly prescribed antibiotic in adults and the most commonly prescribed antibiotic in children (14 vs. 40 patients, respectively). The parenteral route of administration was predominant in both children and adults (93.3% and 96.4%, respectively). Surgical prophylaxis lasting more than one day was the most common indication, often without a specific diagnosis. Medical prophylaxis (the use of antibiotics to prevent bacterial complications of medical conditions) was more commonly practiced in pediatric patients compared with adults (35.9% vs. 17.6%). Justification for antibiotic use was documented in pediatric patients but was largely absent in adult patients. De-escalation was practiced very rarely, with most patients completing the course of antibiotics without any changes to the dose (Table 2).

Patients with HAI were older than those without HAI (42.0 vs. 37.0 years, *p* = 0.305). There was a significant difference in the ward types where HAIs were observed. Specifically, no cases of HAI were identified in the endocrinology, gynecology, hematology, specialized intensive care, neonatology, nephrology, rehabilitation, rheumatology, orthopedics, traumatology, or vascular surgery wards. The majority of patients with HAI were hospitalized in intensive care wards (48.1%), which was significantly different from patients without HAI (Table 3).

Table 4 presents the distribution of HAIs by ward type. Healthcare-associated infections were observed in 27 patients out of 701 (3.8%), with ICU wards accounting for 13 (48.1%) of these cases. In 77.8% of the patients with HAI, the infection was not present at the time of admission, making the current hospital stay the most common source of infection (81.5%). Documented positive microbiological culture results were present in 51.8% of HAIs (14 out of 27 cases), with *Pseudomonas aeruginosa* being the most frequently identified pathogen (5 out of 14 documented cases, 35.7%). Resistance to carbapenems was the most common resistance, followed by resistance to glycopeptides and third-generation cephalosporins.

Surgical-site infections were the most common type of HAI (eight patients, 29.6%), with *P. aeruginosa* identified in two cases, and *Escherichia coli*, *Enterococcus faecalis*, and *Streptococcus pyogenes* identified in one case each (no pathogen was identified in the remaining three cases). Pneumonia was the second most common HAI (seven patients, 25.9%), with *P. aeruginosa*, *Staphylococcus epidermidis*, and other coagulase-negative staphylococci (CNS) identified in one case each, while no pathogen was identified in the remaining four patients. Mild/moderate COVID-19 was the third most common HAI (three patients, 11.1%), with *Acinetobacter baumannii* identified in one case, while no pathogen was identified in the remaining two cases. Urinary tract infections were diagnosed in three patients (11.1%), with no pathogen identified in any of these cases. Sinusitis was observed in two patients (7.4%), both positive for *P. aeruginosa*. Bloodstream infection was identified in a single patient (3.7%) who tested positive for *E. faecalis*. Gastroenteritis was observed in one case (3.7%), with *Enterobacter aerogenes* identified. Skin infection was also observed in a single patient (3.7%), with *Staphylococcus aureus* identified as the causative agent (Table 4).

### 3.2. Study on Antibiotic Consumption in Acute Care Hospitals

Table 5 presents the antibiotics consumed in the same hospitals throughout 2022, disaggregated by ATC5 code, pharmacological group, number of packages consumed, defined daily doses (DDDs), and DDD/100 patient-days. The findings from the PPS and the hospital antibiotic consumption audit were consistent, with ceftriaxone being the most consumed antibiotic, followed by cefazolin. Overall, third-generation cephalosporins were the most consumed pharmacological group, whereas tetracyclines, nitrofuran derivatives, and oxazolidinones had the lowest consumption.

**Figure 2:**
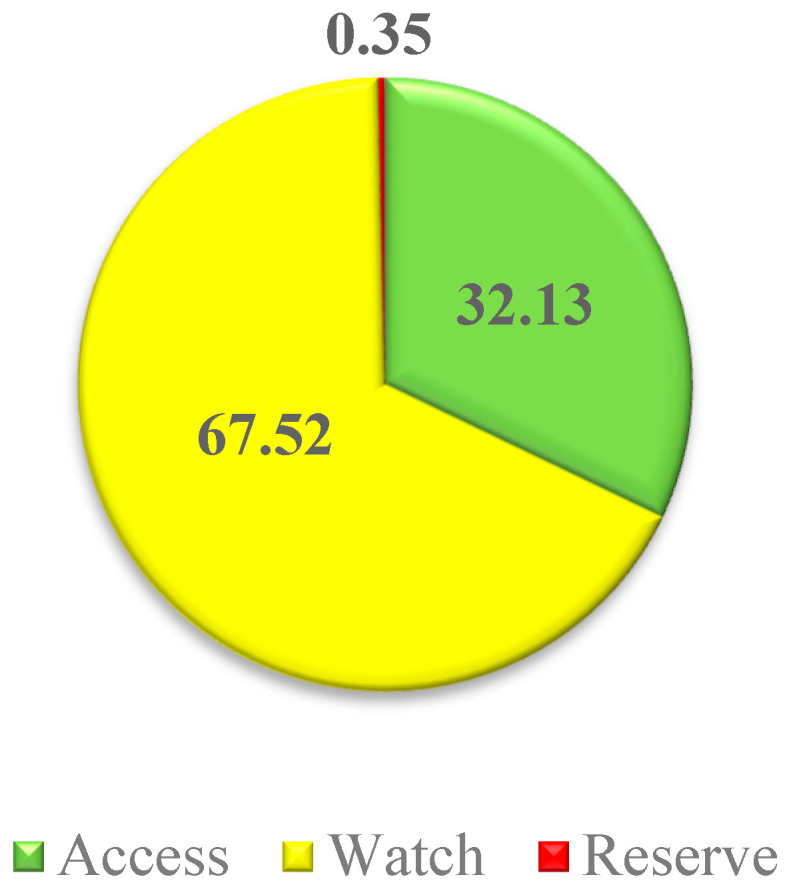
Antibiotic consumption by AWaRe category, %.

Figure 2 supplements Table 5 by providing a visualization of the “Access, Watch, Reserve” (AWaRe) classification categories for the antibiotics consumed. The “Watch” group constituted the largest share, accounting for 67.52%, whereas the “Reserve” group accounted for only 0.35%.

## 4. Discussion

To the best of our knowledge, only one previous survey has investigated the rate of HAIs in Kazakhstan before this PPS was conducted. Viderman et al. reported the HAI rate in the ICU wards of a single oncology center from 2014 to 2015. The reported rate was 19.8% (249/1257 patients), with surgical-site infections being the most common HAI [17]. This PPS showed a slightly lower HAI rate in ICU wards, at 15.1% (13/86 patients), which could be attributed to the earlier period of data collection and the different ward specialties, which were oncology in that case. A common feature in both studies is that surgical-site infections accounted for the majority of HAIs. Another study by Viderman et al. provided insights into the microbiological profiles of HAIs in the same ICU wards. *E. faecalis*, *Klebsiella pneumoniae*, and *P. aeruginosa* were the three most commonly identified pathogens, representing 20%, 15%, and 14% of the cases, respectively. The study also reported high resistance rates to ceftriaxone, cefotaxime, and cefuroxime, as well as significant resistance to carbapenems in *P. aeruginosa* and *A. baumannii* isolates [18]. In this PPS, *P. aeruginosa* was the most common pathogen in ICU wards, followed by *E. faecalis*. Resistance to carbapenems was identified even more frequently than in the study by Viderman et al., possibly reflecting evolving trends in AMR in ICU settings.

In this study, laboratory detection was achieved in only 51.85% of HAIs (14/27), which was generally lower than in other PPS [6,19,20]. Nine different microorganisms were identified, with *P. aeruginosa* being the most commonly isolated pathogen. This is different from other PPSs, which reported a higher prevalence of other pathogens, such as *E. coli* [20], *A. baumannii* [20], and *S. aureus* [19]. *S. epidermidis* is the most common member of the CNS group and is typically found as part of the normal skin flora. However, it can act as an opportunistic pathogen, particularly in immunocompromised patients or those with indwelling medical devices [21]. In a study by Hotterbeekx et al., the potential of *S. epidermidis* to colonize endotracheal tubes and cause ventilator-associated pneumonia was demonstrated [22]. In this study, *S. epidermidis* was identified in a single patient with pneumonia, which could perhaps be explained by the ability of Staphylococci to form biofilms on medical devices [21], allowing them to persist and evade the immune response. In general, the prevalence of different pathogens varies from country to country, between hospitals, and even within the same hospital over time if changes in antimicrobial consumption are observed [3]. This study failed to identify *Clostridium difficile*, which could probably be explained by the small sample size. Clearly, there is a need for more comprehensive studies with larger sample sizes to better understand the distribution of pathogens in different hospital settings in Kazakhstan and to guide targeted interventions for infection prevention and control.

The rate of HAIs observed in acute care hospitals in this study is comparable to that reported in high-income countries, with rates of approximately 4% in the USA [23] and approximately 6.5% in the EU [24]. The rate of HAIs in developing countries is generally higher, averaging 15.5% [25], and a systematic review carried out in Africa revealed that in intensive care unit patients, it ranges between 25.2% and 100% [26]. The reasons behind this elevated rate are related mainly to the lack of infection prevention and control strategies, insufficient infrastructure, and inadequate hand hygiene among healthcare providers [27]. Although the prevention of HAIs is possible in most cases, immunocompromised patients—such as those living with HIV/AIDS, organ transplants, or end-stage cancers—are at increased risk [28].

There are various studies investigating antimicrobial consumption in hospital settings in Kazakhstan [10,11,29], but only one study has examined consumption at the community level [30]. A study by Balapasheva et al. revealed that cephalosporins were the most widely consumed group of antibiotics, accounting for 35.4% of all antibiotics consumed in terms of DDD/100 patient-days [11]. The authors attributed this to the COVID-19 pandemic, which affected the consumption of antimicrobials as well as other classes of medicines [31]. In this study, the share of cephalosporins was even greater, constituting 63.3% of all antibiotics in terms of DDD/100 patient-days, with ceftriaxone alone—a third-generation cephalosporin—accounting for 46.6% of total antibiotic consumption. A study by Makalkina et al. investigating antibiotic consumption in pediatric hospitals also revealed that cephalosporins were the most frequently administered group of antibiotics, with ceftriaxone and ceftazidime ranking the highest [29].

Fluoroquinolones were the second most consumed group of antibiotics in this study, which aligns with the findings of Balapasheva et al., who observed an escalation in fluoroquinolone consumption during the pandemic [11]. Another antimicrobial whose consumption increased during the pandemic is metronidazole [11], and it was among the most consumed antimicrobials in this study as well. Although carbapenems were not among the most consumed antibiotics in this study, most of the isolated microorganisms showed resistance to them. This could be attributed to the fact that carbapenems are mostly administered to patients hospitalized in surgical and ICU wards, where the resistant microorganisms were identified. In general, all previous studies confirmed the observation of this study—that Kazakhstan relies heavily on antibiotics from the watch group, which constitute the bulk of antibiotic consumption in both the hospital [10,11,29] and outpatient healthcare sectors [30]. However, a positive finding is that the consumption of reserve-group antibiotics remains low.

Another common finding across studies investigating antibiotic consumption in Kazakhstan is the route of administration, with parenteral administration being the most prevalent route, even in pediatric practice [29]. This overuse of parenteral administration is a characteristic feature of post-Soviet healthcare systems [9], which persists despite the availability of oral antibiotic formulations on the market. The belief that surgical prophylaxis requires antibiotic administration for more than one day is deeply ingrained among Kazakhstani physicians, contributing to the emergence of resistant strains, even though current evidence suggests that a single dose of antibiotics for surgical prophylaxis can be as effective as multiple doses [32]. There is also a lack of justification for antibiotic prescriptions, particularly in adult practice. Another closely related issue is the lack of antibiotic de-escalation, which in this study was practiced in only 1.1% of cases (3/268), whereas escalation was more common (11.6%, 31/268 patients). This observation is similar to findings from PPS conducted in Brazil [33] and India [34], where a lack of de-escalation strategies was reported. However, antibiotic de-escalation is an important aspect of hospital AMS strategies, as it helps to reduce the risk of developing resistance, and to optimize patient outcomes [35]. Clearly, there is a need for educational interventions and policy changes to address these practices and reduce the risk of AMR in Kazakhstan.

This study has several limitations, the most significant of which is the limited number of hospitals covered by the PPS. As of 2022, when the study was conducted, the total bed capacity in Kazakhstan was 107,214 beds [36], although not all of these are in acute care hospitals. The hospitals included in the study had a total of 2165 beds (2.02% of the total bed capacity in the country), which limits the generalizability of the study findings to the entire nation. However, to address this limitation, a careful hospital selection process was conducted to represent two different cities located in the north and south of Kazakhstan. Additionally, the selection process considered the ward specialties available in different hospitals to ensure a broad range of ward types, covering both pediatric and adult wards. Another limitation was the relatively small number of patients enrolled from the two hospitals located in Almaty, which skewed the sample size toward hospitals in Astana. A possible explanation for this is that daycare treatment is being increasingly implemented in Kazakhstan at different rates in various hospital facilities, which may be the case for the two hospitals sampled in Almaty. This introduces another limitation, as hospital facilities with shorter lengths of stay tend to have lower HAI rates [37]. Another limitation of this PPS is that microbiological results were not available for all patients since the survey was carried out on a single day.

This study also has several strengths. By using an internationally recognized tool and with technical assistance from the country’s WHO office, the study enabled an accurate, internationally comparable assessment of antimicrobial use and HAIs. Another strength lies in the reliability of the data used for analyzing antibiotic consumption at the hospital level, which came from a well-established source frequently used in pharmacoepidemiological research. Future PPS should include other regions of Kazakhstan and a broader range of hospital facilities. There is a need to translate the findings of this study into action and implement infection prevention and control, as well as AMS programs, in hospitals across the country.

## 5. Conclusions

This was the first PPS on antimicrobial use and HAIs in acute care hospitals in Kazakhstan, following an internationally comparable methodology. Overall, the rate of HAIs was low (3.8%) and comparable to that seen in the countries of the EU. Meanwhile, the rate of antimicrobial use was 38.2%, and antimicrobials were often prescribed without adequate justification. Although the findings of this study provide valuable insights into the rates of HAIs and antimicrobial use in acute care facilities in Kazakhstan, they need to be interpreted with caution, as the sample size does not allow for nationwide generalization. A larger PPS is needed to draw more meaningful conclusions; meanwhile, there is a need for AMS strategies in acute care hospitals.

## Figures and Tables

**Table 1:** Distribution of patients by antimicrobial agent consumption (n = 701).

Characteristics		Antimicrobial Agents Not Used (n = 433) N (%)	Antimicrobial Agents Used (n = 268) N (%)	Test of Difference
Age, years	Median (25th; 75th percentiles)	32.0 (12.0; 61.0)	42.0 (18.25; 61.0)	*p* = 0.038 *
Age group, years	<18	154 (35.6)	64 (23.9)	*p* = 0.011 **
	18–29	47 (10.9)	26 (9.7)	
	30–45	73 (16.9)	60 (22.4)	
	46–64	78 (18.0)	67 (25.0)	
	65–75	63 (14.5)	36 (13.4)	
	>75	18 (4.2)	15 (5.6)	
Sex	Male	172 (39.7)	138 (51.5)	*p* = 0.03 **
	Female	261 (60.3)	130 (48.5)	
Type of hospital ward	ICU			*p* < 0.001 **
	Mixed Intensive Care Ward	3 (0.7)	24 (9.0)	
	Other Intensive Care Ward	3 (0.7)	17 (6.3)	
	Medical Intensive Care Ward	6 (1.4)	16 (6.0)	
	Specialized Intensive Care Ward	5 (1.2)	6 (2.2)	
	Pediatric Intensive Care Ward	0 (0.0)	6 (2.2)	
	Medical			
	Neonatology	34 (7.9)	12 (4.5)	
	Hematology	18 (4.2)	12 (4.5)	
	Other Medical Ward	55 (12.7)	12 (4.5)	
	Nephrology	53 (12.2)	7 (2.6)	
	Endocrinology	32 (7.4)	3 (1.1)	
	Rheumatology	14 (3.2)	1 (0.4)	
	Rehabilitation	12 (2.8)	0 (0.0)	
	Surgical			
	Neurosurgery	59 (13.7)	27 (10.0)	
	Other Surgical Ward	4 (0.9)	27 (10.1)	
	Traumatology	10 (2.3)	23 (8.6)	
	Orthopedics	33 (7.6)	20 (7.4)	
	Gynecology	17 (3.9)	13 (4.9)	
	Urology	17 (3.7)	13 (4.9)	
	Burns	6 (1.4)	11 (4.1)	
	Vascular Surgery	10 (2.3)	9 (3.4)	
	Obstetrics/Maternity	42 (9.7)	9 (3.4)	
Type of specialty	Medical	218 (50.3)	48 (17.9)	*p* < 0.001 **
	Surgical	198 (45.7)	151 (56.3)	
	Intensive Care	17 (3.9)	69 (25.7)	

* Test of difference was Mann–Whitney U test. ** Test of difference was Pearson’s chi-square test.

**Table 2:** Comparisons between pediatric and adult patients receiving antimicrobial agents (n = 268).

Characteristics		Children (n = 64) N (%)	Adults (n = 204) N (%)	Test of Difference
Sex	Male	33 (51.6)	99 (48.5)	*p* = 0.672 *
	Female	31 (48.4)	105 (51.5)	
Type of specialty	Medical	21 (32.8)	27 (13.2)	*p* < 0.001 *
	Surgical	20 (31.3)	131 (64.2)	
	Intensive Care	23 (35.9)	46 (22.5)	
Antimicrobial agent (up to 3 agents were administered simultaneously)	Beta-lactams			*p* < 0.001 *
	Piperacillin and tazobactam	7 (8.0)	5 (1.8)	
	Amoxicillin and clavulanic acid	0 (0.0)	5 (1.8)	
	Tazobactam	4 (4.5)	0 (0.0)	
	Aminoglycosides			
	Gentamicin	13 (14.8)	3 (1.1)	
	Amikacin	4 (4.5)	1 (0.4)	
	Fluoroquinolons			
	Ciprofloxacin	6 (6.8)	40 (14.3)	
	Levofloxacin	0 (0.0)	18 (6.5)	
	Ofloxacin	0 (0.0)	2 (0.7)	
	Moxifloxacin	0 (0.0)	1 (0.4)	
	Penicillins			
	Ampicillin	12 (13.7)	3 (1.1)	
	Amoxicillin	1 (1.1)	1 (0.4)	
	Cephalosporins			
	Ceftriaxone	9 (10.2)	99 (35.6)	
	Cefazolin	14 (16.0)	40 (14.3)	
	Cefotaxime	1 (1.1)	8 (2.8)	
	Cefepime	1 (1.1)	4 (1.4)	
	Ceftazidime	2 (2.3)	3 (1.1)	
	Cefuroxime	2 (2.3)	3 (1.1)	
	Ceftriaxone, combinations	1 (1.1)	3 (1.1)	
	Cefatrizine	1 (1.1)	0 (0.0)	
	Cefazedone	0 (0.0)	1 (0.4)	
	Carbapenems			
	Meropenem	7 (8.0)	0 (0.0)	
	Imipenem and cilastatin	0 (0.0)	3 (1.1)	
	Sulfonamides			
	Sulfamethoxazole and trimethoprim	1 (1.1)	1 (0.4)	
	Sulfamethoxazole	0 (0.0)	1 (0.4)	
	Macrolides			
	Spiramycin	0 (0.0)	1 (0.4)	
	Lincosamides			
	Lincomycin	0 (0.0)	1 (0.4)	
	Glycopeptides			
	Vancomycin (parenteral)	0 (0.0)	2 (0.7)	
	Polymyxins			
	Colistin (injection, infusion)	1 (1.1)	2 (0.7)	
	Azoles			
	Fluconazole	1 (1.1)	12 (4.3)	
	Metronidazole (oral, rectal)	0 (0.0)	1 (0.4)	
	Metronidazole (parenteral)	0 (0.0)	14 (5.0)	
Route of administration	Oral	6 (6.7)	9 (3.2)	*p* = 0.403 **
	Parenteral	83 (93.3)	269 (96.4)	
	Rectal	0 (0.0)	1 (0.4)	
Indication	Treatment intention for community-acquired infection	11 (12.4)	70 (25.1)	*p* < 0.001 *
	Treatment intention for infection acquired in long-term care facility or chronic care hospital	5 (5.6)	0 (0.0)	
	Treatment intention for acute hospital-acquired infection	10 (11.2)	39 (14.0)	
	Surgical prophylaxis, single dose	2 (2.2)	12 (4.3)	
	Surgical prophylaxis, one day	2 (2.2)	2 (0.7)	
	Surgical prophylaxis, > 1 day	21 (23.6)	99 (35.5)	
	Medical prophylaxis	32 (35.9)	49 (17.6)	
	Unknown indication	1 (1.1)	8 (2.9)	
	Other indication	5 (5.6)	0 (0.0)	
Diagnoses for which antimicrobial agents were administered	Not applicable; for antimicrobial use other than treatment	63 (72.4)	170 (61.6)	*p* < 0.001 *
	Pneumonia	7 (8.0)	17 (6.2)	
	Surgical site infection involving skin or soft tissue but not bone	0 (0.0)	19 (6.9)	
	Intra-abdominal sepsis including hepatobiliary	0 (0.0)	16 (5.8)	
	Completely undefined, site with no systemic inflammation	6 (6.9)	10 (3.6)	
	Cellulitis, wound, deep soft tissue not involving bone, not related to surgery	0 (0.0)	14 (5.1)	
	Clinical sepsis	6 (6.9)	2 (0.7)	
	Gastrointestinal Infections (salmonellosis, antibiotic associated diarrhea)	0 (0.0)	6 (2.2)	
	Lab-confirmed bacteraemia	0 (0.0)	5 (1.8)	
	Acute bronchitis or exacerberations of chronic bronchitis	0 (0.0)	5 (1.8)	
	Symptomatic lower urinary tract infection	2 (2.2)	3 (1.1)	
	Systematic inflammatory response with no clear anatomic site	2 (2.2)	2 (0.7)	
	Asymptomatic bactreriuria	0 (0.0)	4 (1.4)	
	Septic arthritis, osteomyelitis, not related to surgery	0 (0.0)	2 (0.7)	
	Infections of ear, mouth, nose, throat or larynx	1 (1.1)	0 (0.0)	
	Infections of the central nervous system	0 (0.0)	1 (0.3)	
Reasoning notes	No	3 (3.4)	166 (66.4)	*p* < 0.001 *
	Yes	85 (95.5)	82 (32.8)	
	Unknown	1 (1.1)	2 (0.8)	
Antimicrobial Changed	No	60 (93.8)	173 (85.2)	*p* = 0.013 *
	Escalation	2 (3.1)	29 (14.3)	
	De-escalation	2 (3.1)	1 (0.5)	

* Test of difference was Pearson’s chi-square test. ** Test of difference was Fisher’s exact test.

**Table 3:** Distribution of patients by the presence of healthcare-associated infection (n = 701).

Characteristics		HAI * Is Absent (n = 674) N (%)	HAI * Is Present (n = 27) N (%)	Test of Difference
Age, years	Median (25th; 75th percentiles)	37.0 (14.0; 61.0)	42.0 (18.0; 68.0)	*p* = 0.305 **
Age group, years	<18	213 (31.6)	5 (18.5)	*p* = 0.047 ***
	18–29	67 (9.9)	6 (22.2)	
	30–45	130 (19.3)	3 (11.1)	
	46–64	139 (20.6)	6 (22.2)	
	65–75	86 (12.8)	7 (25.9)	
	>75	39 (5.8)	0 (0.0)	
Sex	Male	295 (43.8)	15 (55.6)	*p* = 0.220 ***
	Female	379 (56.2)	12 (44.4)	
Hospital ward	ICU			*p* < 0.001 ***
	Other Intensive Care Ward	15 (2.2)	5 (18.6)	
	Mixed Intensive Care Ward	23 (3.4)	4 (14.8)	
	Pediatric Intensive Care Ward	3 (0.4)	3 (11.1)	
	Medical Intensive Care Ward	21 (3.1)	1 (3.7)	
	Specialized Intensive Care Ward	11 (1.6)	0 (0.0)	
	Medical			
	Other Medical Ward	64 (9.5)	3 (11.1)	
	Endocrinology	35 (5.2)	0 (0.0)	
	Gynecology	30 (4.5)	0 (0.0)	
	Hematology	30 (4.5)	0 (0.0)	
	Neonatology	46 (6.8)	0 (0.0)	
	Nephrology	60 (8.9)	0 (0.0)	
	Rehabilitation	12 (1.8)	0 (0.0)	
	Rheumatology	15 (2.2)	0 (0.0)	
	Surgical			
	Other surgical	27 (4.0)	4 (14.8)	
	Burns	15 (2.2)	2 (7.4)	
	Neurosurgery	84 (12.4)	2 (7.4)	
	Urology	28 (4.2)	2 (7.4)	
	Obstetrics/Maternity	50 (7.4)	1 (3.7)	
	Orthopedics	53 (7.9)	0 (0.0)	
	Traumatology	33 (4.9)	0 (0.0)	
	Vascular Surgery	19 (2.8)	0 (0.0)	
Type of specialty	Medical	263 (39.1)	3 (11.1)	*p* < 0.001 ***
	Surgical	338 (50.1)	11 (40.7)	
	Intensive Care	73 (10.8)	13 (48.1)	

* Healthcare-associated infection. ** Test of difference was Mann–Whitney U test. *** Test of difference was Pearson’s chi-square test.

**Table 4:** Comparison of patients with hospital-associated infections by ward type (n = 27).

Characteristics		Type of Ward			Test of Difference
		Medical (n = 3), N (%)	Surgical (n = 11), N (%)	Intensive Care (n = 13), N (%)	
Sex	Male	2 (66.7)	6 (54.5)	7 (53.8)	*p* = 0.919 *
	Female	1 (33.3)	5 (45.5)	6 (46.2)	
Age group, years	<18	0 (0.0)	1 (9.1)	4 (30.8)	*p* = 0.571 *
	18–29	0 (0.0)	3 (27.3)	3 (23.1)	
	30–45	0 (0.0)	2 (18.2)	1 (7.7)	
	46–64	1 (33.3)	3 (27.3)	2 (15.4)	
	65–74	2 (66.7)	2 (18.2)	3 (23.1)	
Infection site	Surgical site infection	0 (0.0)	5 (45.5)	3 (23.1)	*p* = 0.045 *
	Pneumonia	2 (66.7)	0 (0.0)	5 (38.5)	
	Mild/moderate COVID-19	0 (0.0)	3 (27.3)	0 (0.0)	
	Urinary tract infection	0 (0.0)	1 (9.1)	2 (15.4)	
	Blood stream infection	0 (0.0)	0 (0.0)	1 (7.7)	
	Sinusitis	0 (0.0)	0 (0.0)	2 (15.4)	
	Gastroenteritis	0 (0.0)	1 (9.1)	0 (0.0)	
	Reproductive tract infections	1 (33.3)	0 (0.0)	0 (0.0)	
	Skin infections	0 (0.0)	1 (9.1)	0 (0.0)	
Infection present at admission	Yes	0 (0.0)	3 (27.3)	3 (23.1)	*p* = 0.589 *
	No	3 (100.0)	8 (72.7)	10 (76.9)	
Origin of the infection	Current hospital	3 (100.0)	10 (90.9)	9 (69.2)	*p* = 0.521 *
	Other acute care hospital	0 (0.0)	1 (9.1)	2 (15.4)	
	Long-term care facility	0 (0.0)	0 (0.0)	2 (15.4)	
Microorganism	*Acinetobacter baumannii*	0 (0.0)	1 (9.1)	0 (0.0)	*p* = 0.161 **
	*Enterobacter aerogenes*	0 (0.0)	1 (9.1)	0 (0.0)	
	*Enterococcus faecalis*	0 (0.0)	0 (0.0)	2 (15.4)	
	*Escherichia coli*	0 (0.0)	1 (9.1)	0 (0.0)	
	*Pseudomonas aeruginosa*	0 (0.0)	1 (9.1)	4 (30.8)	
	*Staphylococcus aureus*	0 (0.0)	1 (9.1)	0 (0.0)	
	*Staphylococcus epidermidis*	0 (0.0)	0 (0.0)	1 (7.7)	
	Other coagulase-negative staphylococci	0 (0.0)	0 (0.0)	1 (7.7)	
	*Streptococcus pyogenesis*	0 (0.0)	1 (9.1)	0 (0.0)	
	Not available	3 (100.0)	5 (45.5)	5 (38.5)	
Antimicrobial resistance phenotype	C3G (Third-generation cephalosporins)	0 (0.0)	2 (18.2)	0 (0.0)	*p* = 0.715 **
	CAR (Carbapenems)	0 (0.0)	4 (36.4)	4 (30.9)	
	GLY (Glycopeptides)	0 (0.0)	1 (9.1)	2 (15.4)	
	OXA (Oxacillin)	0 (0.0)	1 (9.1)	0 (0.0)	
	Not available	3 (100.0)	3 (27.3)	7 (53.8)	

* Test of difference was Pearson’s chi-square test. ** Test of difference was Fisher’s exact test.

**Table 5:** Antibiotic consumption in the four hospitals under study in 2022.

ATC5 Code	Substance	Pharmacological Group	AWaRe * Category	Number of Packages	DDD **	DDD/100 Patient-Days
J01AA02	Doxycycline	Tetracyclines	Access	19	190	0.13347
J01CA01	Ampicillin	Penicillins		10,350	1279.16667	0.89855
J01CA04	Amoxicillin			86	624.666667	0.43880
J01CE01	Benzylpenicillin			7100	1183.33333	0.83123
J01CR02	Amoxicillin and beta-lactamase inhibitor	Beta-lactam		28,179	5317.66667	3.73539
J01CR05	Piperacillin and beta-lactamase inhibitor		Watch	14,514	4187.92857	2.68475
J01DB04	Cefazolin	First-generation cephalosporins	Access	79,540	2,6013.3333	18.27305
J01DC02	Cefuroxime	Second-generation cephalosporins	Watch	11,360	4240	2.97839
J01DD01	Cefotaxime	Third-generation cephalosporins		3700	925	0.64977
J01DD02	Ceftazidime			23,840	5497.5	3.86172
J01DD04	Ceftriaxone			217,380	10,7940	75.82239
J01DD08	Cefixime			50	12.5	0.00878
J01DD12	Cefoperazone			1600	400	0.28098
J01DD52	Ceftazidime and beta-lactamase inhibitor		Reserve	3	10	0.00007
J01DD62	Cefoperazone and beta-lactamase inhibitor			2900	725	0.50928
J01DE01	Cefepime	Fourth generation cephalosporins	Watch	7200	1725	1.21173
J01DH02	Meropenem	Carbapenems		12,528	3942.66667	2.76952
J01DH03	Ertapenem			630	630	0.44254
J01DH04	Doripenem			2152	717.333333	0.50389
J01DH51	Imipenem and cilastatin			3308	827	0.58093
J01EE01	Sulfamethoxazole and trimethoprim	Sulfonamide-trimethoprim combinations	Access	2397	2313.9	1.62540
J01FA09	Clarithromycin	Macrolides	Watch	78	896	0.62939
J01FA10	Azithromycin			208	157.333333	0.11052
J01GB01	Tobramycin	Aminoglycosides		17	85.6	0.06013
J01GB03	Gentamicin		Access	1378	22,966.6667	16.13292
J01GB04	Kanamycin		Watch	50	50	0.03512
J01GB06	Amikacin		Access	5815	4209	2.95661
J01MA01	Ofloxacin	Fluoroquinolons	Watch	1820	910	0.63923
J01MA02	Ciprofloxacin			36,201	9501	6.67397
J01MA12	Levofloxacin			10,703	10,737.5	7.54255
J01MA14	Moxifloxacin			857	857	0.60200
J01XA01	Vancomycin	Glycopeptides		4340	2170	1.52432
J01XB01	Colistin	Polymyxins	Reserve	495	550	0.38635
J01XD01	Metronidazole	Imidazoles	Access	29,835	9977.08333	7.00840
J01XE01	Nitrofurantoin	Nitrofuran derivates		70	175	0.12293
J01XX08	Linezolid	Oxazolidinones	Reserve	602	260.966667	0.18332

* AWaRe—Access, Watch, Reserve Classification. ** DDD—defined daily doses.

## Data Availability

The data presented in this study are available on request from the corresponding author. Due to the personal nature of the data, they are not publicly accessible.
